# Nurse-patient relationship for multi-period home health care routing and scheduling problem

**DOI:** 10.1371/journal.pone.0268517

**Published:** 2022-05-27

**Authors:** Tipaluck Krityakierne, Onkanya Limphattharachai, Wasakorn Laesanklang

**Affiliations:** 1 Department of Mathematics, Faculty of Science, Mahidol University, Bangkok, Thailand; 2 Centre of Excellence in Mathematics, CHE, Bangkok, Thailand; University of Defence in Belgrade, SERBIA

## Abstract

This article proposes a novel dynamic objective function in a multi-period home health care (HHC) problem, known as the nurse-patient relationship (NPR). The nurse-patient relationship score indicating the trust a patient has for his or her care worker increases when the same people meet regularly and decreases when they are apart. Managing human resources in HHC is a combination of routing and scheduling problems. Due to computational complexity of the HHC problem, a 28-day home health care problem is decomposed into daily subproblems, and solved sequentially with the tabu search. The solutions are then combined to give a solution to the original problem. For problems with less complex constraints, the NPR model can also be solved using exact methods such as CPLEX. For larger scale instances, however, the numerical results show that the NPR model can only be solved in reasonable times using our proposed tabu search approach. The solutions obtained from the NPR models are compared against those from existing models in the literature such as preference and continuity of care. Essentially, the analysis revealed that the proposed NPR models encouraged the search algorithm to assign the same care worker to visit the same patient. In addition, it had a tendency to assign a care worker on consecutive days to each patient, which is one of the key factors in promoting trust between patients and care workers. This leads to the efficacy of monitoring patient’s disease progression and treatment.

## 1 Introduction

This study aims to analyze the nurse-patient relationship in Home Health Care (HHC) problem. As a care worker can serve multiple patients in one day, the HHC problem is a combination of routing and scheduling problems. The solution is a daily schedule or a visiting timetable of care workers to meet with the patients at home over a planning horizon (e.g. week, month) [1]. Treating patients at home helps reduce the demand of hospitality infrastructure in a medical center and prevent the risk of infections during the hospital visits. The planning and scheduling become more complicated as the demand increases, especially with the ongoing COVID-19 pandemic in the aging society in which elderly population is growing rapidly.

World Health Organization has projected that by 2050, at least 25 percent of Europe and North America’s population will be older than 65 [2]. The situation does not only exist in the developed countries, but also in developing countries like Thailand [3]. Population projection for Thailand indicated that more than 30.2 percent of the population will be aged 60 and above in 2035. With this statistics, the demand for home health care services will dramatically increase in the near future. The service providers will therefore need efficient decision tools to help them create visit schedules and pairing care workers with patients.

The goal of HHC is to create visiting schedules or plans effectively by considering multiple factors such as travel costs and patients’ satisfaction. HHC problems can be divided into two types by planning horizon. The first type is a single period or a daily problem which is a plan for one day. The other type is a multi-period plan which is a plan for multiple days.

For a single-period model, the problem focuses on finding an integrated routing and scheduling plan. The plan must respect constraints related to patient visiting time window, minimum worker requirements, worker skill qualifications, and patient visit time window [4, 5]. The objective function for a single-period model includes operation costs, travel distances, worker active times, preferences, etc.

For a multiple-period model, the plan puts a higher priority in a scheduling task by finding suitable care workers to meet patients regularly. At the same time, a routing task guarantees that the daily assigned jobs are practical without violating the time window constraints. Several objective functions for a multi-period model have been studied e.g. continuity of care, balancing workload, etc.

To the best of our knowledge, there was no existing research work on evaluating nurse-patient relationship and trust as a level or function. Research in medical science usually collects relationship data from questionnaires or interviews and their analyses focus on trust development and the outcomes of trust that affect medical care [6]. Understanding the relationship between two people is, nonetheless, not straightforward. Defining such a relationship using a mathematical formula can be very complicated, if not impossible. In this work, we propose a concept of nurse-patient relationship. Our study proposes two relationship functions: linear and sigmoidal relationship functions. To reduce the computational complexity, a 28-day HHC problem is separated into subproblems which are then solved sequentially with the tabu search. The performances of the three models, which put priority respectively on the preference, continuity of care, and nurse-patient relationship, are evaluated and compared on 20 modified Cordeau instances.

Thus, the contributions of this paper are twofold. First, we develop for the first time a dynamic model for multi-period home healthcare routing and scheduling problems with nurse-patient relationships where the relationship is evolved and modeled through a linear and sigmoid function. Second, to avoid the model computational complexity associated with the NPR, we propose to solve the multi-period HHC problem by decomposing it into subproblems which are solved sequentially with the tabu search. To verify the effectiveness and efficiency of the proposed tabu search approach, we also attempted to solve subproblems with an exact method, the IBM ILOG CPLEX Optimizer [7]. With the maximum time limit set for each call to an optimizer, we found that the solution returned by CPLEX was not necessarily optimal. Furthermore, numerical results also revealed that, for problem instances with more complex constraints, CPLEX terminated the search without finding a solution. On all problem instances that CPLEX managed to solve, the tabu search could also find the same quality solutions. Moreover, tabu search is capable of solving all 20 problem instances in a reasonable time. This shows that the proposed tabu search approach, while substantially more efficient than CPLEX, is also very robust.

The rest of this paper is organized as follows. Section 2 presents literature reviews. Section 3 gives the definition of a multi-period HHC problem. Section 4 explains our methodology for solving the problem. Section 5 presents experimental results and analysis. Finally, Section 6 gives conclusions and future research directions.

## 2 Literature review

In this section we give the relevant literature review of the home health care problems. Arising in 1980s in order to improve the logistic of home health care, the HHC problem is an extension of the vehicle routing problem (VRP) combining routing and scheduling problems into one [1, 8]. Making decisions by matching care workers with patients, the assigned care workers deliver care services to the patient’s home. The HHC problem differs from the ordinary VRP due to the presence of some features of decision making [9]. This includes, for instance, continuity of care, temporal dependency, preferences, skill/qualification, frequency of visits, etc. Nevertheless, there are some features in VRP that should be considered in the HHC problem such as time-dependent traffic information [10, 11].

Recent research related to the home health care problem has shifted their focus towards assignment factors. A fatigue factor is becoming increasingly important especially during the COVID-19 pandemic, and has been regarded as a crucial objective of the model. Amindoust et al. [12] considered nurse fatigue factors to generate a nurse schedule using a hybrid genetic algorithm. Zhuang and Vincent [13] investigated a nurse scheduling problem with a newly introduced labor law in Taiwan. The changes include a lower limit of weekly working hours, an increment of the minimum interval between two working shifts spanning over two days, and an introduction of a minimum period for inter-work rest. Guo and Bard [14] proposed a column generation-based method to find a midterm nurse schedule. The plan considers nurse preferences and overtime. Sarkar et al. [15] introduced an algorithm for a nurse scheduling problem, where patient recovery is treated as the main objective, and other factors such as nurse skills, and logistic conditions, as constraints. Huang et al. [16] proposed intelligent algorithms to create a personnel schedule to accommodate charge nurses and general nurses whose roster requirements are different under hierarchical management. Z̃iz̃ović et al. [17] suggested a scoring matrix to assign medical professionals to SAR-COV-2 hospitals.

There were several versions of mixed integer programming models for a single period HHC. Trautsamwieser and Hirsch [18] investigated a daily schedule for home health care planning where the problem was formulated using a mathematical model. The single period planning was generated by an exact solver and a variable neighbourhood search (VNS). Their studies showed that a large-scale problem instance cannot be solved by the exact solver within a reasonable time. However, the VNS algorithm was capable of finding feasible solutions for the large-scale problems, and obtaining optimal solutions for smaller problem instances. Laesanklang and Landa-Silva [19] proposed a decomposition method to solve a large-scale real-world workforce scheduling and routing problem. The method started by decomposing the whole problem into multiple subproblems based on geographical regions, and each subproblem was subsequently solved by the exact method. The solutions of subproblems were combined in order to form the solution of the original problem where a repair process may be needed to create a feasible solution. Nasir and Dang [20] proposed a variate neighbourhood search heuristic in order to create a daily home health care plan because a mixed integer programming was unable to solve a large size problem. The goal of their research was to optimize the existing routes while considering compatibility, time restrictions, contract duration, idle time and workload balance. Castaño and Velasco [21] investigated a daily home health care delivery. To find a visit plan while balancing workloads, a network flow-based model and an optimization solver were applied with computation time limit of 3600 seconds to solve a real-world problem in Columbia. Li et al. [22] studied a HHC scheduling and routing problem for outpatient services in the community care center considering time windows, skills, and working regulations as constraints. The problem was formulated as a mixed-integer, nonlinear, convex programming model incorporating travelling costs, waiting time, and patients’ preference objective functions.

Tabu search is one of the most popular metaheuristics algorithms for the home health care problem. Cordeau et al. [23] applied a tabu search algorithm to solve a periodic vehicle routing problem. The method explores the solution space by finding new solutions from the current solution to the best solution in a subset of its neighborhood. This method applied two search operators: remove a customer node from one route and insert it into another route, and replace part of the route with the combination from the other route. Triki et al. [24] applied tabu search algorithm in the two-phase approach for a periodic home health care planning inspired by Cordeau et al. [23]. They applied tabu search to generate a weekly plan and a mixed integer programming based neighbourhood search method to create a daily plan. Gourc et al. [25] used tabu search to solve a single-period multi time-windows home health care scheduling problem. Liu et al. [26] applied a hybridization of tabu search heuristic and local search to solve a periodic home health care problem and it showed promising results. Umam et al. [27] used a hybridization of genetic algorithm and tabu search to find a solution to a flowshop scheduling problem. Schract et al. [28] also utilized a hybrid algorithm between tabu search and genetic algorithm for nurse scheduling problem. Chaieb et al. [29] applied tabu search algorithm to solve the problem of recovery room planning and scheduling during COVID-19 pandemic.

For the multiple period home health care problem, there were many researches in the literature investigating the problem [2, 30]. The key difference between the multi-period problem and the single-period problem is that the patients may request multiple services spread over different days of week or month, and the care workers may work multiple days weekly. The procedure must take into account complex worker assignment and regulations [1].

One important aspect which distinguishes the multiple-period HHC from the single-period HHC is the continuity of care [9]. The continuity of care can be enforced at different levels. The extreme case, or the full continuity of care, happens when the patient must only meet with one care worker during the entire horizon. A relationship between each pair of nurse and patient, measured through the so-called preference score, is also an important factor that should be considered. Wirnitzer et al. [31] tackled a monthly home care rostering problem using a mathematical model where the problem was solved by an optimization solver on a high performance computer with one hour computation time limit. The result showed that none of their models could be solved to optimality within the allotted time, but the best solutions by the optimization solver were better than that from the manual ones. Martinez et al. [32] applied heuristic algorithm combining a MIP formulation and a greedy heuristic to solve HHC problems with continuity of care to create a one-week visiting schedule. Cissé et al. [9] provides a good review of these objective functions.

Table 1 summarizes objective functions from 19 articles that have been used in previous studies of multi-period HHC. From the table, the objective functions in the literature are classified into three groups on the basis of the operations, care workers and patients. Operational factors include travel distance (TD), travel time (TT), total cost (TC), wait time (WT), and number of tasks (TK). Care worker related factors include overtime (OT), work balance (WB), number of care worker (CW), and fairness (FA). Finally, patient related factors include preference (PF), continuity of care (CC), and care worker switches (CS).

**Table 1:** Summary of the existing objective functions for multi-period HHC.

	Operational factors					Care worker factors				Patient factors		
Authors	TD	TT	TC	WT	TK	OT	WB	CW	FA	PF	CC	CS
Guo and Bard (2022) [14]					✓					✓		
Sarkar et al. (2022) [15]				✓		✓				✓		
Schrack et al. (2021) [28]										✓		
Li et al. (2021) [22]										✓	✓	
Castaño and Velasco (2020) [21]			✓				✓					
Martinez et al. (2018) [32]		✓		✓			✓			✓	✓	
Wirnitzer et al. (2016) [31]								✓			✓	✓
Duque et al. (2015) [33]	✓									✓		
Bowers et al. (2015) [34]										✓	✓	
Bard et al. (2014) [35]	✓		✓			✓						
Carello and Lanzarone (2014) [36]						✓					✓	
Nickel et al. (2012) [2]	✓				✓	✓				✓	✓	
Barrera et al. (2012) [37]							✓	✓				
Bachouch et al. (2011) [38]	✓											
Trautsamwieser and Hirsch (2011) [18]		✓				✓			✓	✓		
Bennett and Erera (2011) [39]									✓		✓	
Ikegami and Uno (2007) [40]				✓			✓	✓				
Steeg and Schröder (2007) [30]								✓		✓		
Begur et al. (1997) [41]		✓										

Shorthand notation: travel distance (TD), travel time (TT), total cost (TC), wait time (WT), number of tasks (TK), overtime (OT), work balance (WB), number of care worker (CW), fairness (FA), preference (PF), continuity of care (CC), care worker switches (CS)

From these objectives, the preferences, the continuity of care and care worker switches reflect directly the quality of services and patient satisfaction [34]. Most home health care providers require continuity of care as a condition of their roster to avoid potential loss of information when a patient meets a new care worker. Receiving care from the same worker also ensures good service quality. There were several researches in the literature implementing continuity of care. Steeg and Schröder [31] proposed a model to minimize the number of care workers to visit a patient considering nurse-patient loyalty as the main indicator for continuity of care. Nickel et al. [2] defined patient-nurse loyalty as the number of additional care workers assigned to a patient. If the patient-nurse loyalty is equal to zero, then the patient is assigned to only one care worker which is the best possible solution. Carello and Lanzarone [36] proposed a model to minimize the cost associated with reassignments. They defined a binary variable of reassignment for each patient and each time slot, and the goal is to find a solution with the least number of different care workers per patient. The reassignment variable is equal to 1 if the assignment to the current time slot of the patient is changed from the previous time slot, and 0 otherwise. So if the patient is assigned to the same care worker at every time slot, the number of reassignment for the patient will be zero which is the best possible solution. Ikegami and Uno [40] investigated the home help staff scheduling problem with workload balance to equalize working hours amongst all care workers. Unlike the proposed nurse-patient relationship (NPR) model which evolves daily, none of these objective functions for HHC appearing in previous work are dynamic.

**Fig 1:**
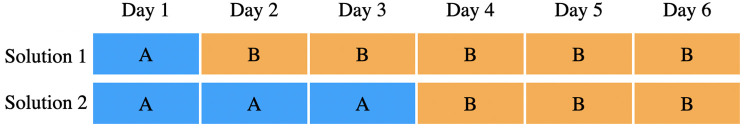
Example of two distinct solutions with the same continuity of care for one patient over 6 days.

As the continuity of care was defined as the number of different care workers assigned to one patient, the model attempts to minimize the number of workers assigned to a patient. Let us remark that it is possible to have two distinct solutions with the same continuity of care. Fig 1 presents two example schedules for one patient, each having two care workers, A and B, visiting a patient for six days. Evaluating the two schedule with continuity of care will result in the same score, as both plans assign two workers. However, in reality, Solution 1 should have a higher score because Worker B met the patient for five consecutive days. To resolve this issue, we propose a novel dynamic nurse-patient relationship function which continues to evolve and is updated daily with prior assignments. To the best of our knowledge, this is the first study using a dynamic function within the framework of home health care routing and scheduling problems.

## 3 Problem definition

A multi-day HHC makes assignments for care workers to serve patients at their home for multiple days in the planning horizon. Depending on the patient’s condition, each patient may or may not need a visit everyday. It is also possible to have requests that are spread as specific day-of-the-week patterns. Care workers may also have days-off. All patient requests and care worker days-off during the planning horizon are known at the time a decision is made. Therefore, our goal is to create a multi-day plan for HHC that best matches a care worker with patients considering their restrictions and availabilities. The plan also takes into account travelling distances. Nevertheless, the priority is to provide service quality for patients through preference scores, continuity of care, or nurse-patient relationship.

Let us summarize now the conditions required for our model formulation.

1. Patients request dates and times of the visits.
2. All patient requests must be met.
3. Care workers may make multiple visits in one day.
4. Visits must be made during care worker availability. No overtime assignments are allowed.
5. Assignments must prioritize the care worker who is more familiar with the patient, preferably the one who made a frequent visit.
6. Assignments should guarantee that care workers can make a visit where the travel time must be taken into the decision process.
7. It is preferable to assign care workers with a higher preference score. However, a lesser preferred care workers can be assigned if no other care workers are available.

All these aspects will be investigated thoroughly in this work. We now propose mathematical programming models for HHC.

### 3.1 Sets and parameters

A multi-period home health care problem described in this work can be defined over a graph *G* = (*V*, *E*), where *V* = {0, 1, …, *n*} and *E* = {(*i*, *j*)|*i*, *j* ∈ *V*, *i* ≠ *j*} represent the vertices and the edges of the graph where *i*, *j* ∈ {0, 1, …, *n*}, respectively. The depot, denoted by 0 is the initial location of all care workers, a vertex *i* ∈ *V*∖{0} represents a patient’s home, and *n* is the number of patients. In addition, *C* denotes the set of all care workers and *D* denotes the set of days in the problem planning horizon. Table 2 provides the list of notations used in this paper.

**Table 2:** List of notations.

Set description	Notation
set of locations	*V*
depot (care workers’ starting location)	0 ∈ *V*
set of patients	*V*′ = *V*∖{0}
set of care workers	*C*
set of days	*D*
Parameter description	Notation
travel distance between patient *i* and patient *j*	*α*_*ij*_
travel time from patient *i* to patient *j*	*β*_*ij*_
time window (earliest & latest time) for care workers to visit patient *j* on day *d*	$\left[e_{j}^{d},l_{j}^{d}\right]$
duration of service time for visiting patient *j*	*γ*_*j*_
preference score between care worker *c* and patient *j*	$p_{j}^{c}$
service requested by patient *j* on day *d*	$\delta_{j}^{d}$
time availability (ready time and due date) for care worker *c* on day *d*	[*σ*^*cd*^, *ϵ*^*cd*^]
parameter indicating on duty for care worker *c* on day *d*	*ζ* ^*cd*^
travel distance weight	*w*_1_
preference weight	*w*_2_
continuity of care weight	*w*_3_
NPR relationship weight	*w*_4_
a sufficiently large value	*M*

### 3.2 Variables

The MIP models proposed in this work have two sets of decision variables given in (1) and (2). Define a binary variable $x_{ij}^{cd}$ to be an assignment for care worker *c* that travels from patient *i* to patient *j* on day *d* and a non-negative integer variable $a_{j}^{cd}$ to be the arrival time of care worker *c* at patient *j* on day *d*:

$$\begin{array}{c} x_{ij}^{cd}=\{\begin{array}{cc} 1, & \;\text{if}\;\text{edge}\;\left(i,j\right)\;\text{is}\;\text{assigned}\;\text{to}\;\text{care}\;\text{worker}\;c\\ & \text{on}\;\text{day}\;d\\ 0, & \;\text{otherwise} \end{array} \end{array}$$ (1)

$$\begin{array}{cc} a_{j}^{cd}\in Z^{0+}, & \text{the}\;\text{time}\;\text{care}\;\text{worker}\;c\;\text{arrives}\;\text{at}\;\text{patient}\;j\;\\ & \text{on}\;\text{day}\;d\text{.} \end{array}$$ (2)

### 3.3 Constraints

$$\begin{array}{cccc} \sum_{i\in V}x_{ij}^{cd} & =\sum_{k\in V}x_{jk}^{cd} & & \forall j\in V^{\prime },d\in D,c\in C \end{array}$$ (3)

$$\begin{array}{cccc} \sum_{j\in V^{\prime }}x_{0j}^{cd} & =\sum_{i\in V^{\prime }}x_{i0}^{cd}\leq 1 & & \forall d\in D,c\in C \end{array}$$ (4)

$$\begin{array}{cccc} \sum_{i\in V}\sum_{c\in C}x_{ij}^{cd} & =\delta_{j}^{d} & & \forall j\in V^{\prime },d\in D \end{array}$$ (5)

$$\begin{array}{cccc} \sum_{i\in V}\sum_{j\in V^{\prime }}x_{ij}^{cd} & \leq M\zeta^{cd} & & \forall c\in C,d\in D \end{array}$$ (6)

$$\begin{array}{c} e_{j}^{d}\sum_{i\in V}x_{ij}^{cd}\leq a_{j}^{cd}\leq l_{j}^{d}\sum_{i\in V}x_{ij}^{cd}\;\;\forall j\in V,d\in D,c\in C \end{array}$$ (7)

$$\begin{array}{c} a_{i}^{cd}+\gamma_{i}+\beta_{ij}-a_{j}^{cd}\leq \left(1-x_{ij}^{cd}\right)M\;\;\forall i,j\in V,d\in D,c\in C \end{array}$$ (8)

$$\begin{array}{c} \sigma^{cd}\leq a_{i}^{cd}\leq \epsilon^{cd}\;\;\;\;\;\forall i\in V,d\in D,c\in C \end{array}$$ (9)

$$\begin{array}{ccc} x_{ij}^{cd}\in \left\{0,1\right\} & & \forall i,j\in V,d\in D,c\in C \end{array}$$ (10)

$$\begin{array}{ccc} a_{j}^{cd}\in Z^{0+} & & \forall j\in V,d\in D,c\in C \end{array}$$ (11)

Constraint (3) guarantees the coherence of each route, i.e. if care worker *c* visits patient *j*, then the care worker must leave that visiting location. For constraint (4), a route must begin and end at the depot only once each day.

Constraint (5) guarantees that all patient requests are served where $\delta_{j}^{d}$ is a binary parameter equal one if patient *j* requests a service on day *d*, and zero otherwise. Constraint (6) ensures that assignments on day *d* are assigned to care workers who are on duty on day *d*. Note that the on-duty parameter *ζ*^*cd*^ is also a binary parameter, equal one if the care worker *c* is available on day *d*, and zero otherwise. Constraint (7) prevents time window violations. Constraint (8) assigns arrival time to visit patient *j*. Constraint (9) guarantees that the arrival time $a_{j}^{cd}$ is within the care worker availability.

### 3.4 Objective function

In this work, we consider and compare three models, namely, Basic, continuity of care (CC), and nurse-patient relationship (NPR).

#### 3.4.1 Basic model

In the Basic model, we minimize a linear combination of the total travel distances and the preference costs. The objective function is given by (12). The total travel distances are a summation of distances computed from all care workers while the total preference costs are a summation of the preference score contributed from all care workers. The higher the preference score, the lower the objective function value.

$$\begin{array}{c} f_{Basic}=\sum_{d\in D}\sum_{c\in C}\sum_{i,j\in V}w_{1}\alpha_{ij}x_{ij}^{cd}-\sum_{d\in D}\sum_{c\in C}\sum_{i,j\in V}w_{2}p_{j}^{c}x_{ij}^{cd} \end{array}$$ (12)

#### 3.4.2 Continuity of care (CC) model

Built upon *f*_*Basic*_, the continuity of care model considers the maximization of continuity of care in addition [1, 31]. The equivalent minimization problem is given by (13). The term relating to the continuity of care cost simply counts the total number of care workers that serve each patient. In this case, if a patient is served by the same care worker, the objective value is lower.

$$\begin{array}{c} f_{CC}=f_{Basic}+\sum_{c\in C}\sum_{j\in V}w_{3}y_{j}^{c} \end{array}$$ (13)

The MIP model for this case requires an additional parameter which is a continuity of care weight *w*_3_. Also, a binary variable $y_{j}^{c}$ is defined to be 1 if the care worker *c* has been assigned to visit patient *j* at least once, as shown in (14)–(16).

$$\begin{array}{c} y_{j}^{c}=\{\begin{array}{cc} 1, & \;\text{if}\;\text{care}\;\text{worker}\;c\;\text{has}\;\text{been}\;\text{assigned}\;\text{to}\;\text{visit}\\ & \;\text{patient}\;j\;\text{at}\;\text{least}\;\text{once}\\ 0, & \;\text{otherwise} \end{array} \end{array}$$ (14)

$$\begin{array}{ccc} \sum_{i\in V}\sum_{d\in D}x_{ij}^{cd}\leq My_{j}^{c} & & \forall j\in V,c\in C \end{array}$$ (15)

$$\begin{array}{ccc} y_{j}^{c}\in \left\{0,1\right\} & & \forall j\in V^{\prime },c\in C \end{array}$$ (16)

#### 3.4.3 Nurse-patient relationship (NPR) model

*(1) NPR with linear relationship function and its limitations*. To capture the acquaintance level, we introduce a variable $\tau_{j}^{c,d}$ that increases as the care worker *c* regularly meets the patient *j*, and decreases otherwise. Based on the artificial pheromone in ant colony optimization algorithm [42], $\tau_{j}^{c,d}$ is computed using the formula in (17), where *Q* is a growth constant and *ρ* is a decay rate of the relationship when a care worker has not met a patient for several days:

$$\begin{array}{c} \tau_{j}^{c,d}=\{\begin{array}{cc} \tau_{j}^{c,d-1}+\left(Q\times p_{j}^{c}\right), & \text{if}\;\text{care}\;\text{worker}\;c\;\text{meets}\\ & \text{patient}\;j\;\text{on}\;\text{day}\;d\\ \left(1-\rho \right)\tau_{j}^{c,d-1}, & \text{otherwise,} \end{array} \end{array}$$ (17)

where the initial relationship score is zero, i.e. $\tau_{j}^{c,0}=0,\forall j\in V,c\in C$.

Let us remark that despite its simplicity, there are some theoretical limitations with the linear NPR function. First, since the linear function has no bound, the linear NPR term grows without bound as we move forward in the planning horizon, making it difficult to control the weights of objective functions. This also prevents one from carrying the NPR relationship into the next planning horizon. Also, another limitation of the linear relationship is the strict assumption of linearity of the relationship over time which may not necessarily hold true for the relationship.

*(2) NPR with sigmoidal relationship function*. The NPR with sigmoidal relationship framework, introduced in this section, is extensible to the next planning horizon and possesses some desired properties. Essentially, not only do sigmoid curves increase over time (with bound), they also represent the law of diminishing marginal utility which states that the marginal utility of a service declines as more of it is received by an individual.

The sigmoidal relationship score is derived as the summation of the nurse-patient relationship score of care workers to be deployed on each day. The solution whose assignments are made with a higher nurse-patient relationship score will have a lower objective function value. This is represented by the additional term of the objective function in (18).

$$\begin{array}{c} f_{NPR}=f_{Basic}-\sum_{d\in D}\sum_{c\in C}\sum_{j\in V}w_{4}f\left(\tau_{j}^{c,d}\right)x_{ij}^{cd} \end{array}$$ (18)

where *w*_4_ is a relationship weight, $\tau_{j}^{c,d}$ quantifies the level of acquaintance, and $f(\tau_{j}^{c,d})$ is a relationship score between care worker *c* and patient *j* on day *d*.

Our assumption for the nurse-patient relationship score is that the relationship level will increase when a care worker visits a patient repeatedly. In contrast, if the care worker is absent or other care workers meet the patient, the relationship with the absent care worker is deteriorating. Moreover, the relationship score should increase slowly during the first few visits, and increase more rapidly once the care worker and patient get better acquainted. Finally, the relationship value should not exceed some predefined upper bound. We thus assume that our nurse-patient relationship follows the sigmoid function or S-curved [43].

To this end, we define the relationship score $f(\tau_{j}^{c,d})$, with the zero lower bound (the lowest relationship score) and the upper bound of 1 (the highest relationship score) as shown in (19) where *k* is a slope, and *b* is a shifting constant:

$$\begin{array}{c} f\left(\tau_{j}^{c,d}\right)=\frac{1}{1+\text{exp}\left(-k\times \left(\tau_{j}^{c,d}-b\right)\right)}. \end{array}$$ (19)

Note that for the linear relationship function, the function $f(\tau_{j}^{c,d})$ in (18) is simply the identity map.

Let us now dive into discuss time complexities of the NPR models with sigmoid and linear functions. There is no doubt that the sigmoid function will require a longer computation time compared to the linear function. It is known however that using the floating point exponentiation, the complexity is always O(1). For this reason, there is not a significant difference between the time complexity for the two relationship functions, be it linear or sigmoid. In particular, the overall complexity of a typical step of the algorithm is dominated by the procedure used to find the solutions in the search space (Algorithm 3), therefore the difference in the computation time required for the two relationship functions can in fact be negligible. Empirical evidence that shows the computation time to solve NPR models with sigmoid and linear relationship functions are not significantly different can be found in Table 6.

### 3.5 Comment: Computational complexity of the multi-period HHC problem

A single-period home health care problem is classified as an NP-hard problem [2]. Thus, the multi-period home health care problem is also an NP-hard problem with additional dimensions for the planning periods. Our mathematical programming model for the multi-period problem has two sets of decision variables: a set of binary variable $x_{ij}^{cd}$ which allocates visits for care worker *c* to visit patient *j* after patient *i* on day *d*, and a set of variable $a_{j}^{cd}$ which captures visiting time for care worker *c* when visiting patient *j* on day *d*. Considering only these two sets of variables, the total number of decision variables in the objective function is

$$\left|C\right|\cdot \left|D\right|\cdot {\left|V\right|}^{2}+|C|\cdot |D|\cdot |V|=|C|\cdot |D|\cdot |V|\cdot (|V|+1).$$

Solving the problem as a whole, the search space for the binary variables is $2^{\left|C\right|\cdot \left|D\right|\cdot {\left|V\right|}^{2}}$ combinations. Here, the number of combinations is referred to as the maximum number of subproblems generated during the branch-and-bound operations in conventional MIP solvers. Solving the problem considering all time periods at once also requires impractically large amounts of memory. In particular, for the multi-period HHC model, the memory required for the LP relaxation could take up to 1 GB. Obviously, the amount of memory is increased during branch-and-bound operations as the number of potential subproblems due to branching can be as many as $2^{\left|C\right|\cdot \left|D\right|\cdot {\left|V\right|}^{2}}$.

In contrast, considering the problems day-by-day concerns only $2^{\left|C\right|\cdot {\left|V\right|}^{2}}$ combinations per subproblem. The memory required here in this case is significantly lower than that required to solve the problem as a whole. In particular, the memory required to solve each subproblem is approximately 100 MB for the LP relaxation, where the maximum number of branch-and-bound subproblems is $2^{\left|C\right|\cdot {\left|V\right|}^{2}}$ subproblems.

It was evident that using optimization solvers may require extremely high computational resources. For example, Wirnitzer et al. [31] used high performance computing with 121 GB of Ram to solve a problem consisting of 37 nurses, 143 patients, a total of 1114 visits for 7 days with 3 shifts per day. This vast amount of resources is not available in a personal computer. Thus, we propose in the next section to tackle this by decomposing the multi-period problem into multiple daily subproblems, reducing significantly the computational requirements. While mixed integer nonlinear programming solvers exist in the literature, such algorithms do not guarantee optimality when tackling a non-convex function, and a metaheuristic such as tabu search has more popularity for solving this problem.

## 4 Methodology

In this section, we present a method to solve multi-period HHC problems. Although solving the complete set of multi-period problem as a whole guarantees finding the optimal solution, the method is very computationally expensive as discussed. To reduce the computational complexity, the method given in Algorithm 4 separates the problem into |*D*| subproblems where |*D*| is the number of days in the multi-day problem. The algorithm solves the subproblems sequentially and generates |*D*| sub-solutions. The solutions of the |*D*| subproblems are then combined to give a solution to the original problem.

**Algorithm 1**: Main algorithm

**Input**: Problem *P*

**Output**: Solution *S*

1: Divide problem *P* into |*D*| subproblems *P* = {*P*_1_, *P*_2_, ⋯, *P*_|*D*|_}

2: *P*_*d*_ = a subproblem containing patients and workers for day *d*

3: *S*_*d*_ = a solution to the subproblem *P*_*d*_

4: Set initial $\tau_{j}^{c,0}=0$ and $f(\tau_{j}^{c,0})=0$

5: Initialize *S* as Empty

6: **for** *P*_*d*_ = *P*_1_, ⋯, *P*_|*D*|_ **do**

7:  *S*_*d*_ ← *initSol*_*d*_ from Greedy Algorithm (Algorithm 2)

8:  *S*_*d*_ ← *FinalSol*_*d*_ from Tabu Search (Algorithm 3)

9:  Append *S*_*d*_ to *S*

10:  Update $f(\tau_{j}^{c,d})$ using (19)

11: **end for**

12: **return** *S*

For each subproblem, the method generates an initial solution using a greedy algorithm, and the tabu search algorithm is then used to improve the solutions. The assignments made by the tabu search will be lead by the three models presented in Section 3.4. For the NPR model, after the sub-solution for a 1-day assignment is improved, the relationship scores are updated, following the procedure described in the previous section, before the process to solve the subsequent subproblem begins. In this way, the relationship score can be viewed as a parameter of the subproblem. The processes are repeated until all subproblems are solved.

### 4.1 Initial solution

**Algorithm 2**: Greedy algorithm

**Input**: Problem *P*_*d*_: $L_{d}$ (a list of patients left to assign on day *d*), $f(\tau_{j}^{c,d}$)

**Output**: Initial solution *initSol*_*d*_

1: Initialize *initSol*_*d*_ as Empty

2: **while** $L_{d}\neq \left\{\right\}$ **do**

3:  **for** $l_{j}\in L_{d}$ **do**

4:   Assign the available care worker to patient *l*_j_*

5:   **if** (The assignment does not violate constraints) **then**

6:    Update solution *initSol*_*d*_

7:    Update $L_{d}$: remove *l*_*j*_ from $L_{d}$

8:   **end if**

9:  **end for**

10: **end while**

11: **return** *initSol*_*d*_

* For the Basic model, assign the available care worker with highest preference score.

* For the CC model, assign the available care worker who has previously visited patient *l*_*j*_ and with the highest preference score.

* For the NPR model, assign the care worker with highest nurse-patient relationship score.

Our method starts by generating an initial solution using the greedy algorithm. The overall procedure is presented in Algorithm 2 with some specific details for each model. For the Basic model, the algorithm assigns an available care worker with highest preference score. For the CC model, the algorithm assigns a care worker who has been assigned previously and with highest preference score. Finally, the NPR model assigns a care worker with highest nurse-patient relationship score. In all three models, this assignment made in the initial solution must not violate any constraints, otherwise the next available care worker with highest preference score (for Basic and CC) or highest relationship score (for NPR) is assigned, given that the assignment does not violate any constraints. The procedure is repeated until all patients are assigned.

### 4.2 Tabu search

After generating an initial solution, the solution is improved by tabu search. This procedure is presented in Algorithm 3. Behaving like a local search algorithm, tabu search accepts also non-improving solutions to escape from a local optimum trap [44]. A key feature of the tabu search is the use of temporary memory, called tabu list, which records solutions that have previously been visited. A long length of the tabu list creates diversification search which explores wider solution regions and forbids the algorithm to revisit solutions already examined. A shorter length of the tabu list intensifies the search in the area that is deemed to have good solutions. During the search, the algorithm requires search operators to generate new solutions from existing solutions [45]. The operator applied in this work is shift (1,0) move—relocating a customer from one route to another.

**Algorithm 3**: Tabu search

**Input**: Fitness function *F*, Problem *P*_*d*_, Initial solution *initSol*_*d*_

**Output**: Solution *FinalSol*_*d*_

1: *S* ← *initSol*

2: *BestF* = *F*(*S*)

3: Initialize *tabu list* as Empty

4: **while** *i* < *I*_*max*_ or *i*_*nonimprove*_ < *IMax*_*nonimprove*_ **do**

5:  Select two routes *r*_1_, *r*_2_ in *S* randomly

6:  Apply Shift(1,0) to *r*_1_, *r*_2_, finding the best neighborhood solution *S*′ which is neither in *tabu list* nor violates constraints

7:  *S* ← *S*′ and update *tabu list*

8:  Set *i*+ = 1

9:  **if** *F*(*S*′)<*BestF* **then**

10:   *FinalSol*_*d*_ ← *S*′

11:   *BestF* = *F*(*S*′)

12:   *i*_*nonimprove*_ = 0

13:  **else**

14:   Set *i*_*nonimprove*_+ = 1

15:  **end if**

16: **end while**

17: **return** *FinalSol*_*d*_

## 5 Computational experiments

In this section, we present experiments using the proposed method to solve modified benchmark problems. The algorithms were written in Python and tested on a machine with Intel Core i5 CPU @ 2.3GHz, 8GB RAM. Parameters configurations and problem instances are presented in Sections 5.1 and 5.2, respectively. The results of the three models are given in Section 5.3. The last section is devoted to an analysis of the effects of applying a relationship score.

### 5.1 Parameter setting

For the proposed NPR model, parameters for the benchmark problems are specified as follows. Parameters *ρ* and *Q* in the NPR function are decay rate and growth constant of the relationship. A high decay rate *ρ* reduces the relationship score faster if a care worker is absent. Also, a high growth constant *Q* increases the relationship score more when a care worker makes a frequent visit to the same patient. The parameters *k* and *b* are chosen to create a sigmoid function with range in (0, 1). While these parameters can be adjusted to accommodate different levels of relationship, Table 3 summarizes the values of the model parameters used in our numerical experiments. The values of weights assigned to each objective function are also given in the same table.

**Table 3:** Model parameters used in numerical experiments. The parameter *α*_0_ = max{*α*_0,*j*_} is the maximum distance between the depot 0 and patient locations *j* of each instance.

Parameter	*ρ*	*Q*	*k*	*b*	*w*_1_	*w*_2_	*w*_3_	*w*_4_
Value	0.2	1	3	2	1	*α*_0_	2 × *α*_0_	2 × *α*_0_

The highest priority objective function for the HHC problem is the term related to the continuity of care (*w*_3_ of *f*_*CC*_) or the nurse-patient relationship (*w*_4_ of *f*_*NRP*_). The second most important objective is the preference objective (*w*_2_). Finally, the lowest priority objective is the term related to total travel distance (*w*_1_). In order to prioritize the importance of each objective function, we follow the idea given in [8, 19]. Here, we set the preference weight *w*_2_ equal to the distance between the service center and the furthest location. We set *w*_3_ in the CC model equal to 2 × *w*_2_ so that the weighted sum objective function will prioritize the continuity of care over the preference score. Finally, the weight *w*_4_ in the NPR model is also twice the weight of the preference score *w*_2_. The parameter *α*_0_ = max{*α*_0,*j*_} is the maximum distance between the depot 0 and patient locations *j* of each instance.

### 5.2 Instances

Instances for this research are based on Cordeau instances which were originated for the classical VRPTW. Cordeau instances are stored on the website of VRP-REP [46] dedicated to the study of VRPs. The original Cordeau datasets have 20 different instances. We use the instances consisting of 25 patients and 25 care workers. The following procedure is carried out to transform the Cordeau instances into the ones appropriate for HHC.

1. Generate preference scores between every pair of care workers and patients. The range of preference score is 0 to 1. Higher scores indicate a higher preference level. A non-preferred care worker with score -1 may also be assigned to a patient if no preferred care workers are available on some days.
2. Create a multi-day instance with periodic demands. The original instance is modified to a multi-day instance with mixed periodic demands. Periodic demands mean the patients reserve cares in a pattern such as every week, every two weeks, etc. Thus, each request is distributed in the pattern of the same day-of-week.

The information of periodic instances consists of the number of patients who want to be served and the number of available care workers on each day as presented in Table 4. The first column represents the instance name, the other columns represent the day index. The two rows represent the number of the patients who want to be served on each day (#Patient), and the number of available care workers (#Worker), respectively.

**Table 4:** Information of modified problem instances.

Instance		Day																											
		1	2	3	4	5	6	7	8	9	10	11	12	13	14	15	16	17	18	19	20	21	22	23	24	25	26	27	28
pr01	#Patient	8	10	10	10	8	11	13	9	9	11	10	7	13	12	8	9	8	11	8	14	11	9	10	10	12	9	14	11
	#Worker	12	13	14	15	14	9	11	13	13	15	15	14	8	11	12	13	14	16	14	9	11	12	13	14	13	14	7	9
pr02	#Patient	12	8	9	9	10	9	12	16	11	9	9	9	12	9	11	12	10	10	9	13	11	14	12	10	9	8	9	11
	#Worker	13	14	14	15	11	14	16	14	14	13	15	10	14	16	13	13	14	15	10	13	15	14	14	14	15	11	13	14
pr03	#Patient	10	12	10	10	7	15	9	10	12	10	10	9	15	10	11	11	9	10	7	11	9	9	9	9	11	9	17	7
	#Worker	14	11	10	16	12	11	9	13	11	10	16	12	11	9	13	11	8	17	12	11	10	13	10	11	17	13	11	10
pr04	#Patient	12	10	7	10	9	10	14	14	10	8	9	9	11	11	14	9	8	6	11	10	12	13	9	8	10	7	11	13
	#Worker	16	13	11	14	15	12	15	16	13	10	12	14	12	16	16	12	11	14	16	12	14	17	10	10	14	15	12	16
pr05	#Patient	11	13	10	8	7	12	16	9	11	11	8	8	9	14	12	10	8	8	10	7	15	12	12	10	8	11	10	13
	#Worker	13	13	14	13	9	11	12	11	14	14	13	8	11	12	13	15	14	11	10	11	12	12	13	12	12	8	11	12
pr06	#Patient	11	14	6	11	12	10	11	12	11	8	10	10	8	12	12	14	8	9	11	10	9	10	15	8	12	12	9	11
	#Worker	11	16	15	13	9	14	11	10	14	13	13	10	17	12	10	16	15	14	9	17	12	11	16	13	14	10	17	12
pr07	#Patient	14	10	9	12	7	10	13	13	11	8	10	8	11	14	12	12	8	9	5	12	15	9	12	12	11	7	13	14
	#Worker	15	9	13	12	11	11	10	15	9	13	13	11	13	11	14	9	10	12	11	13	11	13	9	10	13	10	13	9
pr08	#Patient	11	11	6	8	11	13	9	7	11	6	9	12	11	9	9	11	5	9	14	11	8	8	11	7	9	10	14	10
	#Worker	12	12	13	13	15	13	14	13	13	14	12	17	14	14	13	12	13	12	17	14	14	11	12	14	12	17	14	12
pr09	#Patient	11	12	10	10	9	10	13	15	12	7	7	6	11	12	15	12	9	9	9	8	15	11	11	8	10	10	8	14
	#Worker	15	11	14	12	12	12	12	15	11	13	12	11	12	12	15	11	12	12	12	12	12	15	12	14	12	13	12	12
pr10	#Patient	10	11	7	12	7	11	12	9	9	9	11	8	13	7	9	7	5	8	9	11	14	7	9	6	11	9	11	9
	#Worker	14	15	15	12	16	12	13	14	15	15	14	15	13	15	14	14	15	14	16	13	13	14	14	14	14	15	13	15
pr11	#Patient	11	7	11	5	13	13	8	11	8	8	8	16	12	9	9	7	12	9	15	10	10	10	10	11	7	14	11	10
	#Worker	11	14	12	18	12	11	11	11	14	12	18	12	15	11	11	13	11	18	13	13	10	12	13	12	19	13	13	11
pr12	#Patient	12	11	15	14	9	10	12	12	10	10	14	9	11	8	14	11	11	14	8	9	12	10	10	12	14	10	9	10
	#Worker	11	15	13	7	9	11	13	10	15	13	8	12	12	12	11	14	14	7	12	12	13	10	15	14	7	10	12	13
pr13	#Patient	9	12	10	6	8	9	13	10	11	11	9	8	8	13	9	12	11	8	9	7	11	10	10	6	7	9	6	11
	#Worker	10	10	15	15	7	10	14	12	10	14	15	8	10	15	13	10	13	13	8	10	14	12	8	14	14	8	8	15
pr14	#Patient	7	4	5	9	13	12	9	9	6	7	5	12	12	8	8	6	10	9	12	12	8	9	5	9	7	11	5	10
	#Worker	8	15	15	10	13	11	12	9	14	15	10	13	11	12	7	15	15	10	13	10	13	9	13	14	9	13	11	13
pr15	#Patient	14	12	13	9	8	17	5	12	11	10	10	8	14	6	10	10	12	10	11	16	7	12	11	14	10	11	15	7
	#Worker	15	10	15	11	12	12	11	14	10	14	13	12	13	12	13	11	14	13	13	12	13	13	10	15	13	13	12	12
pr16	#Patient	8	10	12	7	8	8	11	8	10	12	11	9	5	12	12	11	11	12	9	7	9	10	12	7	13	8	8	7
	#Worker	11	10	12	9	12	10	9	10	11	11	8	14	10	9	11	9	10	8	14	11	9	10	11	11	9	14	9	8
pr17	#Patient	12	10	12	14	10	8	5	13	11	13	14	13	9	9	12	8	12	15	12	8	8	13	10	13	13	12	8	7
	#Worker	13	13	14	14	16	14	12	13	14	12	13	15	14	12	15	12	13	12	16	14	9	14	13	13	14	16	15	12
pr18	#Patient	7	8	10	7	14	10	11	5	8	11	10	13	10	12	8	6	10	11	13	10	11	6	8	10	10	11	11	13
	#Worker	8	9	12	18	14	8	12	6	10	12	17	14	9	11	7	10	11	16	14	9	12	8	9	11	18	14	8	11
pr19	#Patient	13	7	8	9	10	8	6	11	12	9	9	11	9	8	12	13	10	8	10	9	7	11	13	8	6	11	10	9
	#Worker	13	15	12	17	10	12	10	12	14	12	15	10	12	9	13	15	12	16	11	10	10	13	15	12	17	11	12	10
pr20	#Patient	8	10	9	11	12	10	6	9	10	7	13	10	8	8	9	10	9	13	10	10	7	11	8	8	14	10	9	7
	#Worker	15	12	13	10	11	14	11	14	11	14	10	9	13	12	15	10	14	10	10	13	12	15	11	14	10	11	13	12

### 5.3 Overall results

This section presents results obtained from the Basic, CC, and the two NPR relationship models. Since the results of the NPR models with linear (NPR(L)) and sigmoid (NPR(S)) relationships were not significantly different, we will discuss them together as the NPR models hereafter, and defer comments on their similarities to Subsection 5.3.1.

To compare the quality of the solutions found by our solution method, an alternative approach which employs the simplex algorithm was implemented to solve the linear NPR subproblems. In particular, the effectiveness of our approach is tested against the state-of-the-art IBM ILOG CPLEX Optimizer [7]. We shall refer to this variant as NPR(L)-Cpx. However, in order not to interrupt the flow of the presentation, we will postpone the discussion of the comparisons with NPR(L)-Cpx to Subsection 5.3.2.

Table 5 presents the value of the four objectives, namely, the total travel distance (Distance), the total preference score (Preference Score), the number of different care workers (Diff Worker) and the total relationship score (Relationship Score). The comparison of the four models can be done by comparing the objective function value evaluated at the optimal solution returned by the model. Therefore, even if the model itself does not involve a particular objective function, one could still compute the objective function value at the optimal solution, see e.g. Wirnitzer et al. [31]. Here, we use the sigmoidal function as the measurement of the relationship score.

**Table 5:** The objective function values of the solution obtained from the five models. The symbol ↓ indicates the lower the objective value the better, while ↑ indicates the opposite.

Instance	Distance ↓					Preference Score ↑				
	Basic	CC	NPR(L)-Cpx	NPR(L)	NPR(S)	Basic	CC	NPR(L)-Cpx	NPR(L)	NPR(S)
pr01	31564.38	32985.98	30743.3	34792.82	34093.12	227.69	226.63	224.99	232.3	224.61
pr02	31158.64	33542.22	31237.3	34204.66	34204.66	247.8	245.45	242.82	240.06	240.06
pr03	26476.88	27747.5	23470.9	28811.98	28811.98	228.34	228.58	219.28	235.6	235.6
pr04	30453.28	32217.76	29511.8	32956.5	32956.5	238.59	242.66	240.22	237.6	237.6
pr05	25883.66	26239.94	25052.0	28001.56	28001.56	236.46	239.59	232.03	240.68	240.68
pr06	33757.94	35899.54	33384.5	37066.84	36300.66	231.41	237.66	237.20	235.72	240.23
pr07	36783.38	37258.94	35867.5	38009.04	38009.04	248.73	238.61	245.91	245.62	245.62
pr08	27258.96	29518.56	26762.1	29022.88	29022.88	221.85	224.72	215.32	222.98	222.98
pr09	37023.44	38733.58	34243.1	40079.16	39237.5	241.1	250.06	241.63	250.6	247.58
pr10	26489.58	27613.66	24960.9	28774.3	27716.06	211.12	214.9	210.41	218.58	212.57
pr11	30850.28	32947.68	N/A	33867.94	34403.44	246.05	253.74	N/A	252.63	252.52
pr12	33765.84	35944.64	N/A	37305.5	36698.36	238.26	231.46	N/A	227.6	226.54
pr13	27243.68	27705.06	26238.5	29474.94	29188.48	210.31	201.47	204.38	210.62	210.64
pr14	28681.4	28258.08	27887.2	31582.28	31413.28	189.79	175.7	183.10	192.14	191.54
pr15	26520.72	29080	N/A	30332.68	30332.68	232.84	247.92	N/A	249.29	249.29
pr16	27444.26	29540.24	N/A	31470.62	31907.78	209.87	213.25	N/A	226.56	225.18
pr17	35817.36	35233.6	N/A	37817.78	37817.78	261.81	256.72	N/A	264.87	264.87
pr18	25373.34	26513.82	N/A	28234.48	26959.26	227.84	226.92	N/A	228.7	221.35
pr19	35620.8	36350.4	32240.29	38535.28	38014.58	214.73	203.81	207.63	224.81	220.73
pr20	27646.08	28390.54	27253.51	30701.28	29995.74	214.24	214.76	214.91	222.07	219.95
average	30290.695	31586.087	29203.79*	33052.126	32754.267	228.9415	228.7305	222.84*	232.9515	231.507
Instance	Diff Worker ↓					Relationship Score ↑				
	Basic	CC	NPR(L)-Cpx	NPR(L)	NPR(S)	Basic	CC	NPR(L)-Cpx	NPR(L)	NPR(S)
pr01	85	55	52	56	54	83.14	106.45	119.19	115.20	119.25
pr02	78	52	58	54	54	115.11	138.04	142.39	139.03	139.03
pr03	91	61	67	59	59	84.80	114.18	116.32	121.49	121.49
pr04	86	59	60	56	56	91.48	112.37	131.73	120.22	120.22
pr05	85	64	63	59	59	89.17	107.49	127.96	125.93	125.93
pr06	93	56	58	53	55	81.86	121.34	136.71	132.06	136.61
pr07	80	58	65	59	59	103.70	127.61	127.23	138.35	138.35
pr08	84	50	49	51	51	72.29	110.05	126.01	127.98	127.98
pr09	100	63	61	60	65	78.58	105.35	119.35	124.87	116.64
pr10	85	45	57	50	46	79.33	113.67	108.91	124.31	124.08
pr11	82	56	N/A	53	52	107.45	134.48	N/A	149.20	153.72
pr12	95	57	N/A	59	58	73.62	109.30	N/A	119.91	120.22
pr13	82	57	63	58	59	62.14	74.15	80.78	88.46	88.55
pr14	79	49	51	52	54	50.95	66.91	74.80	74.75	73.90
pr15	106	63	N/A	60	60	86.51	109.56	N/A	129.75	129.75
pr16	106	67	N/A	67	66	63.62	89.00	N/A	100.85	96.42
pr17	79	58	N/A	53	53	130.30	141.44	N/A	163.38	163.38
pr18	82	57	N/A	55	55	64.26	93.48	N/A	105.29	99.74
pr19	99	62	66	61	59	73.00	91.65	96.55	104.89	104.35
pr20	95	57	59	55	54	69.46	97.16	110.04	107.85	109.21
average	88.6	57.3	59.21*	56.5	56.4	83.04	108.18	115.56*	120.69	120.44

* N/A values excluded from the calculation

Considering the first column, we can see that the Basic model required the fewest number of trips while the NPR models required the largest number of trips. Looking at the column “Distance”, overall the Basic model performs better than the other two, which came as no surprise as the basic model gives priority to minimizing the distance. As for “Preference Score”, the three models achieve similar results, but the average value for the NPR is highest. As for “Diff Worker”, it is also apparent that the total number of different care workers required for the CC and NPR models are less than that of the Basic model. Finally, the results in the column “Relationship Score” clearly show that the NPR models have highest relationship score for all instances while the Basic model has the lowest relationship score for all instances.

Table 6 displays computational times (in seconds) to find a solution for each instance. Due to complexity of the relationship score, the NPR models required significantly more computation time than the other two models.

**Table 6:** Computation time (seconds).

Instance	Model variants				
	Basic	CC	NPR(L)-Cpx	NPR(L)	NPR(S)
pr01	11	17	3497	348	331
pr02	10	15	3451	314	293
pr03	11	19	3062	358	294
pr04	8	17	2826	291	257
pr05	11	19	2933	345	290
pr06	10	16	2829	443	295
pr07	11	18	3132	335	326
pr08	8	14	3023	266	277
pr09	9	16	3262	295	327
pr10	8	13	2722	253	266
pr11	11	22	N/A	383	429
pr12	14	24	N/A	480	527
pr13	11	22	15307	446	485
pr14	10	17	8400	358	410
pr15	11	22	N/A	407	544
pr16	12	23	N/A	412	554
pr17	10	19	N/A	396	460
pr18	13	22	N/A	431	493
pr19	11	18	15053	390	490
pr20	11	19	18758	374	473
Average	10.55	18.6	5512.85*	366.25	391.05

* N/A values excluded from the calculation

**Fig 2:**
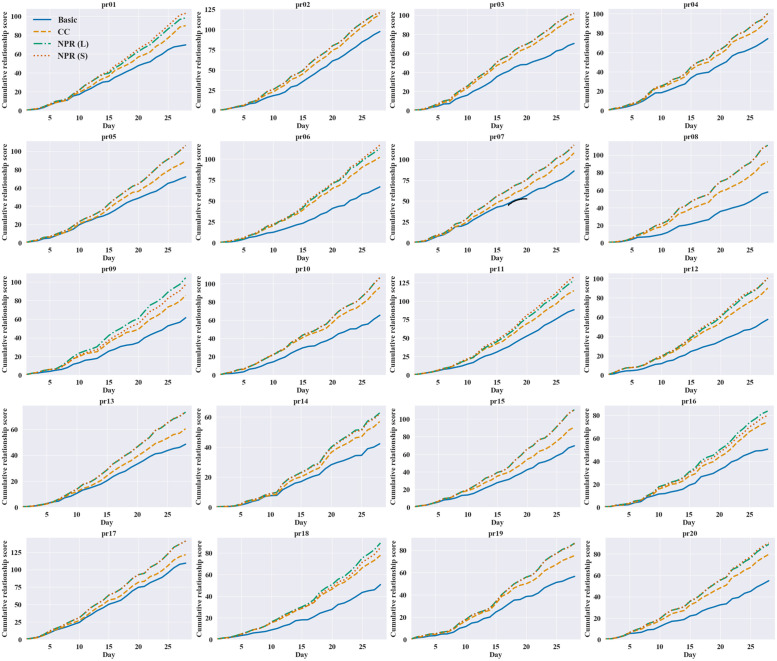
Cumulative total relationship scores over the 28-day horizon.

Additionally, we examine daily cumulative relationship scores of each instance. Fig 2 presents the cumulative total relationship scores of the four models over the planning horizon. We can see from the graphs that NPR models have highest cumulative relationship scores overall, followed by the CC, and Basic models, respectively.

#### 5.3.1 Comparison between the NPR models with linear and sigmoid relationship functions

Results for the two NPR models in Table 5 indicated that the NPR model with sigmoid and linear functions each achieved the best NPR score on 6 problems, and were tied on 8 problems. Thus, based on our results, the NPR models are indifferent to relationship function choice. This conclusion is later supported by a statistical test in Section 5.3.3. Taking a look at the computation times for the two NPR models, as presented in Table 6, it appears there is no significant difference. These results confirm our discussions regarding the time complexities of the two relationship functions in Section 3.4.3. It is worth reiterate that despite having nearly the same performance on this benchmark suite, practically the sigmoidal NPR is more flexible than the linear one due to its extensibility to the next time horizon.

#### 5.3.2 Comparing the tabu search-based with the CPLEX-based approaches

The objective of this section is to determine the quality of the relationship scores found with the tabu search heuristic. When solving the subproblems, the alternative method is based on the simplex algorithm, CPLEX. As we found no statistically significant difference between the linear and sigmoid NPR models, we will only consider solving the linear NPR model with the CPLEX algorithm.

Table 5 gives the objective function values of solutions found by the CPLEX-based approach. In particular, results of the relationship score (last panel of the table) reveal that the solutions of the linear NPR model found by the CPLEX (NPR(L)-Cpx) and those by the tabu search (NPR(L)) are quite similar for instances with less complex constraints. With the maximum time limit set for each call to an optimizer, we could see that the solution returned by CPLEX is not necessarily optimal. Moreover, for relatively complex instances (pr11–12, 15–18), NPR(L)-Cpx failed to solve the problem and terminated the search without finding a solution within maximum time limit of 1800 seconds per subproblem.

**Fig 3:**
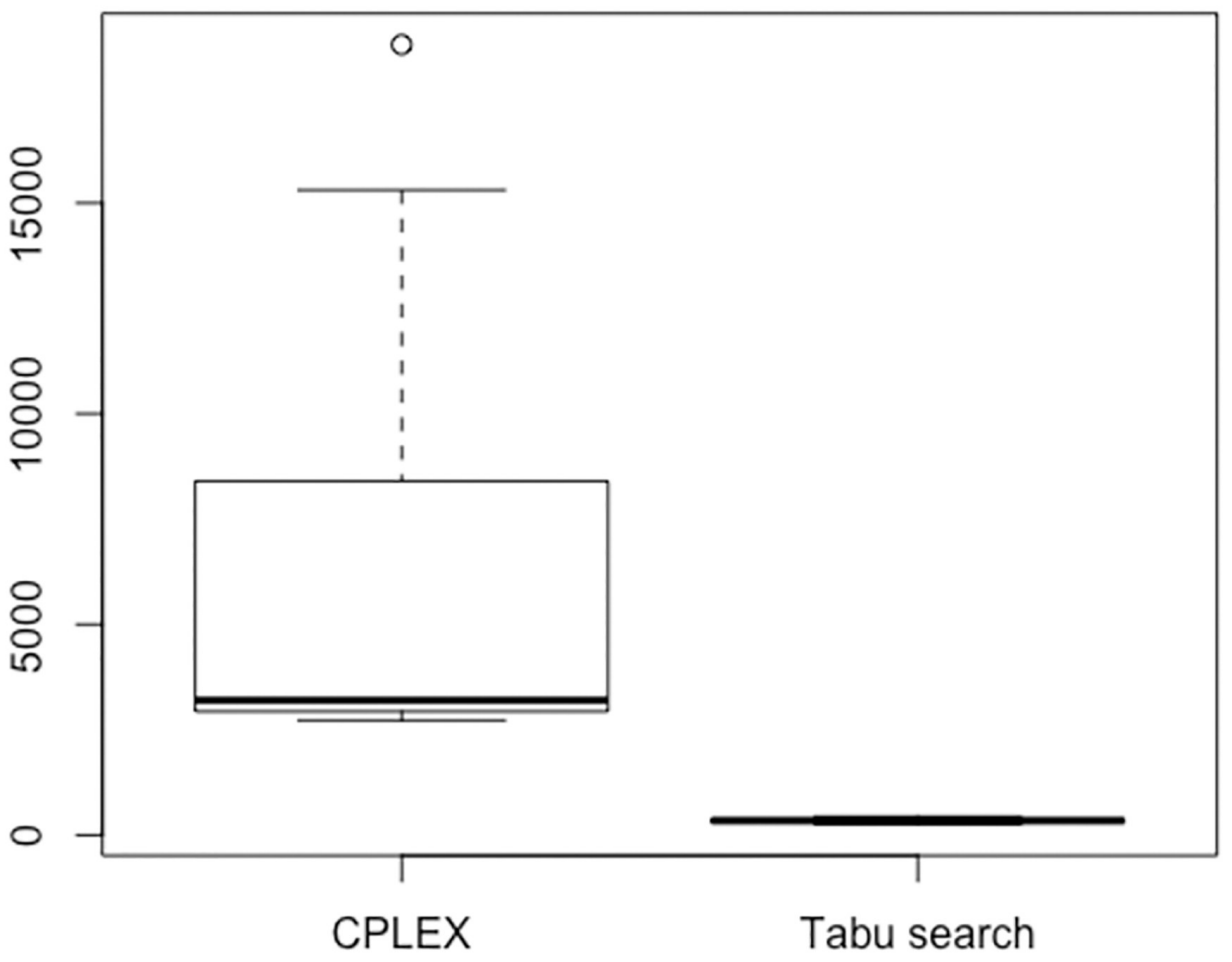
Computational time of CPLEX versus tabu search (seconds). Problems with N/A values are excluded.

Table 6 and Fig 3 give the computation time of NPR(L)-Cpx versus NPR(L). It reveals that for instances with less complex constraints, the linear NPR model could also be solved using CPLEX. The computation time of CPLEX, however, increases significantly as the problem complexity increases. Consequently, for larger scale instances (in terms of constraints) the model can only be solved in a reasonable time with the proposed tabu search method.

Furthermore, the computation time of our proposed tabu search approach scales very well with the instance complexity, while CPLEX does not. In particular, calculating the improvement in the algorithm runtime (by taking the quotient of the runtimes NPR(L)-Cpx over NPR(L)), we obtained the ratio which ranges between 6x and 50x. This means our approach could achieve at least 6-fold runtime improvement over CPLEX. (For problems that CPLEX could not find a solution, this ratio is infinity).

#### 5.3.3 Statistical tests on the relationship score obtained from the five models

Statistical tests have been carried out to see whether the obtained values of the relationship score found by the five model variants are significantly different. First, the obtained relationship scores of each model were aggregated in Table 7 by the mean-rank method. Note that when we calculated the mean rank, we only include the 14 problem instances for which the NPR(L)-Cpx algorithm could find a solution. A Friedman test was subsequently carried out to compare the results of the relationship scores achieved by each model variant.

**Table 7:** Mean rank of the models based on the relationship scores, conducted on the 14 problem instances without N/A values. The higher the value, the better the model.

Model	Mean rank
Basic	1.00
CC	2.14
NPR(L)-Cpx	3.86
NPR(L)	4.00
NPR(S)	4.00

As the Friedman test results given in Table 8 is significant, we further conducted a Wilcoxon signed-rank test to locate which pairs are significantly different at the 5% level of significance. The test results are summarized in Table 9. Indeed, the test found no significant difference between the linear and sigmoid NPR models. The test found also no significant difference between the linear models with CPLEX and the tabu search (N/A values removed from the calculation). NPR models are significantly better than the Basic and CC models. Finally, the Basic and CC models are not significantly different.

**Table 8:** Friedman test results of the five models on the relationship scores.

N	14
Chi-square	42.745
df	4
*P*-value	<0.001

**Table 9:** Pairwise comparison with a Wilcoxon signed-ranks test.

Hypothesis	*P*-value
Basic vs. CC	.278
Basic vs. NPR(L)-Cpx	<.001*
Basic vs. NPR(L)	<.001*
Basic vs. NPR(S)	<.001*
CC vs. NPR(L)-Cpx	.041*
CC vs. NPR(L)	<.001*
CC vs. NPR(S)	<.001*
NPR(L)-Cpx vs. NPR(L)	>.999
NPR(L)-Cpx vs. NPR(S)	>.999
NPR(L) vs. NPR(S)	>.999

* Asterisk indicates that two groups are significantly different at significant level 0.05.

### 5.4 Observations of effects of the nurse-patient relationship score

Besides achieving a good relationship score, this section aims at illustrating the indirect effects of the NPR models through observations. While other objectives, namely, total distance and total preference score also influence the optimization results of the NPR models, as the models give priority to the relationship score, we present two easy-to-understand solutions to highlight the NPR effects on 1) minimizing the number of care workers and 2) promoting consecutive assignments.

**Fig 4:**
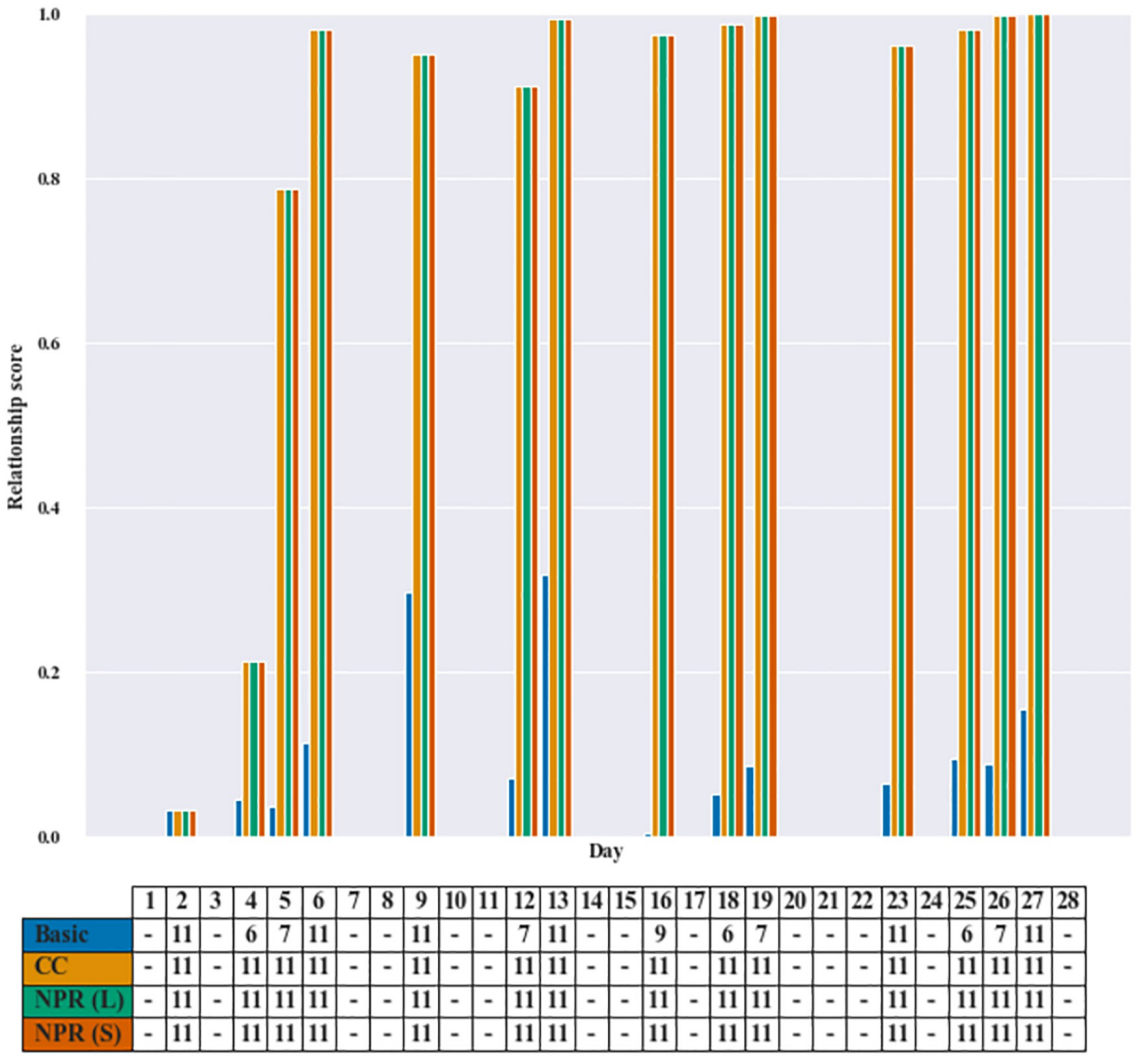
Instance pr18—Patient 19. The bar graph illustrates the relationship score of Patient 19 with the designated care worker ID given in the table.

**Fig 5:**
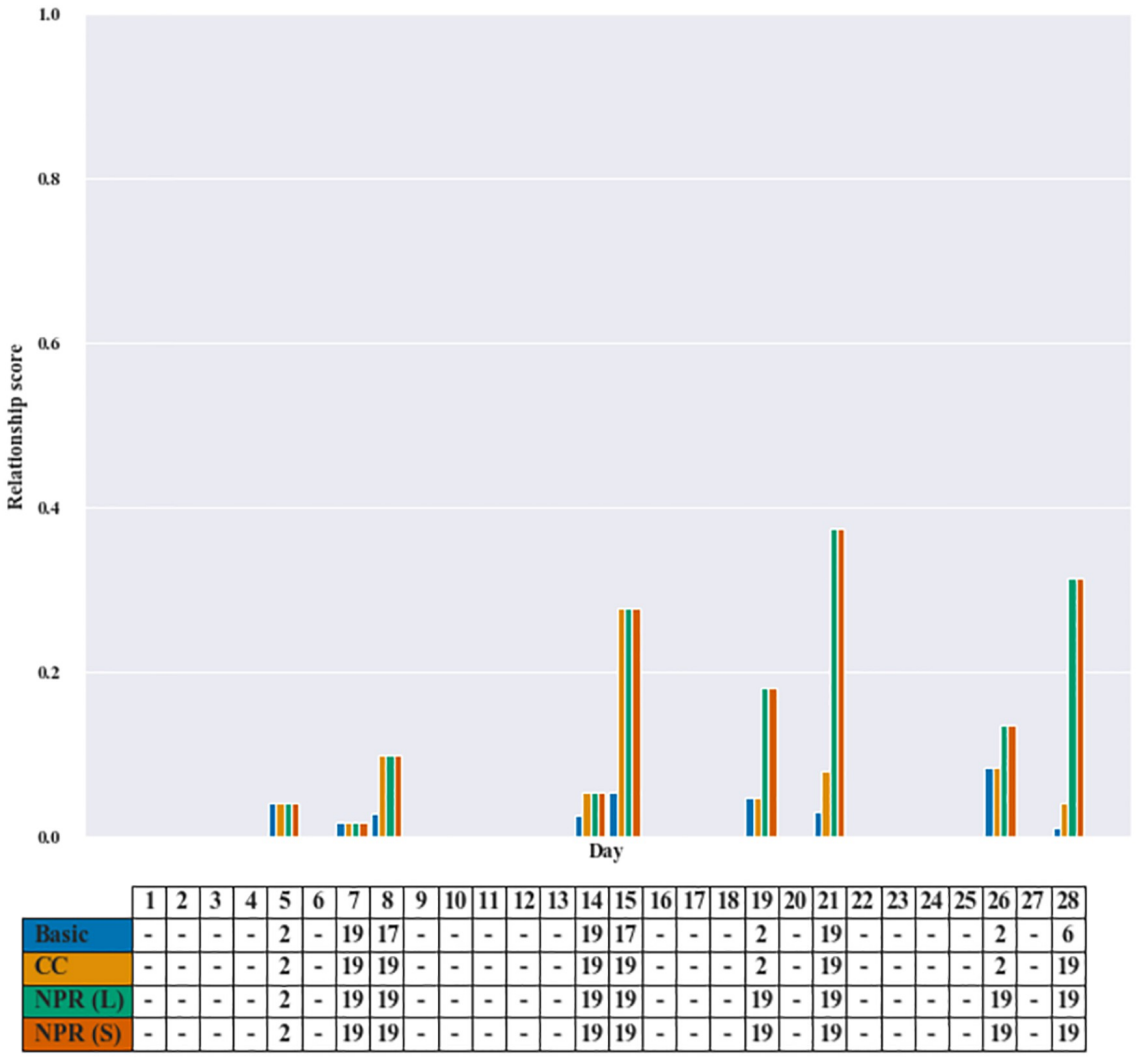
Instance pr01—Patient 18. The bar graph illustrates the relationship score of Patient 18 with the designated care worker ID given in the table.

Figs 4 and 5 give a bar graph comparing the relationship score and an assignment table of a representative patient. Each figure consists of two parts: a bar graph and its corresponding table. The vertical axis represents the relationship score of the patient with the assigned care worker, and the horizontal axis represents the day index. The schedule table in the figure shows assignments where the column corresponds to day in the planning horizon, and the three rows give the care worker ID assigned to the patient. Note that if there is no request from a patient on any given day, then we do not display the value of the relationship score in the bar graph, and the assignment is marked by a dash (-).

#### 5.4.1 Minimizing the number of care workers: Instance #pr18 [Patient 19]

The first example illustrated in Fig 4 focuses on the assignment pattern for Patient 19 of instance pr18. The result shows that the relationship scores of NPR and CC models for Patient 19 with a designated care worker are higher than that of the Basic model on each day. Also, from the table we can see that both CC and NPR models assign only one care worker (Care worker 11) to this patient over the entire horizon. On the other hand, since the Basic model gives priority to the preference score, each day the model tends to select the available care worker with highest preference score without taking into account the continuity of care nor relationship. This results in four different care workers visiting Patient 19 over the entire horizon.

Recall that the CC model gives priority to minimize the number of care workers visiting a patient. While the priority of the NPR models is to maximize the nurse-patient relationship score, our results reveal that to achieve that goal the model simultaneously minimizes the number of care workers.

#### 5.4.2 Promoting consecutive assignments: Instance #pr01 [Patient 18]

The second example given in Fig 5 focuses on the assignment made for Patient 18 as a part of solutions for instance pr01. The results showed that the relationship score of NPR on each day is highest. In addition, the assignment pattern indicates that NPR models have a tendency to assign care workers on consecutive days, while the other two models do not.

We see also that CC and NPR models both assign only Care workers 2 and 19 to the patient (despite being assigned in different order). Thus, the total number of different care workers for Patient 18 of CC and NPR models are both equal two care workers. Results on Day 19 however reveal different assignments made by the two models—the CC model assigns Care worker 2 because of a higher preference score (the preference score for Care worker 2 with Patient 18 is $p_{18}^{2}=0.941$ while the preference score for Care worker 19 with Patient 18 is $p_{18}^{19}=0.633$). This leads to a lower relationship score of CC when compared to NPR on Day 19 as shown in the graph. For the NPR models, they keep assigning Care worker 19 to the patient because Care worker 19 has been assigned to this patient previously for four consecutive times, thus having higher relationship score. This clearly demonstrates the essence of NPR as a function promoting consecutive assignments which should be one of the key indicators of quality of care—as using the same care worker can lead to the efficacy of monitoring patient’s disease progression and treatment.

Let us remark that these direct observations of the effects of NPR score were exemplified by a fixed patient, fixed problem instance over the entire horizon. We are not attempting to generalize these observations to all patients by any means.

## 6 Conclusion and future work

The continuity of care model proposed in the previous studies of the multi-period HHC attempts to reduce the number of different care workers assigned to each patient without taking into account the consecutive assignments of the same care worker. Because of this, the CC model does not promote the consecutive assignments of the same care worker to meet with the patients. To systematically improve the CC model, this article presents a novel nurse-patient relationship model. The dynamic nurse-patient relationship score captured by the NPR models demonstrate the trust a patient has for the care worker when they regularly meet. The model focuses on enhancing patient satisfaction by assigning a care worker with highest nurse-patient relationship score to the patient. To the best of our knowledge, there was no existing research work that examines a dynamic relationship function within the framework of HHC.

Extensive experimentation has been carried out. Twenty instances of the 28-day HHC problem are solved using the proposed heuristic algorithm combining a greedy algorithm and tabu search. Furthermore, to test the efficiency of the proposed approach, CPLEX has been used as an alternative solver to solve subproblems with less complex constraints. However, numerical results showed that CPLEX could not handle several problem instances with complex constraints, terminating the search without finding a solution. Tabu search, on the other hand, coped well with all problem instances. In addition, tabu search required almost the same computation time across all problem instances. The overall results indicated, in addition, that the NPR models improved care worker utilization, achieving highest relationship score for all instances. Moreover, not only the NPR models attempted to reduce the number of care workers assigned to a patient, we found also that the model promoted consecutive assignments arranging the same care worker to meet with the patient recently visited.

While our findings are important and promising, as a future work, there is still room for improving the sigmoid model to create a scoring system to reflect, capture closer to the real-world nurse-patient relationship, and investigate the consequences of applying the nurse-patient relationship to the problem with other objective functions such as workload balance, number of tasks, fairness, etc. In addition, when dealing with uncertain scheduling problems, it will be interesting to consider the robust optimization for HHC planning as unplanned absenteeism among care workers might present especially during the pandemic due to the risk of COVID-19 infection.

In terms of limitations, although we have showed that the tabu search method could achieve much faster convergence than CPLEX when solving the NPR model, Table 6 reveals a drawback of the proposed NPR model compared to the CC model. It is evident from the table that computational complexity of the NPR models is a major bottleneck of practical implementation when trying to extend the model to a longer-horizon multi-period model. Another limitation is the fact that the trust level in the proposed NPR model only relies on the frequency of visits by a particular care worker. In reality, however, there are many scenarios that could contribute to relationship breakdown, e.g. work stress and burn out, nurse unfit to perform a job for a health condition, no call—no show at work, and several other possible causes. As these negative factors can cause a decrement in trust, modeling a more flexible relationship function that can incorporate other factors leading to the creation and/or breakdown of trust will be interesting avenues for future research.

## Supporting information

S1 Dataset (ZIP)
